# Correction: Classical and Bayesian estimation for type-I extended-F family with an actuarial application

**DOI:** 10.1371/journal.pone.0307200

**Published:** 2024-07-10

**Authors:** The PLOS One Staff

There is an error in affiliation 3 for author Omid Kharazmi. The correct affiliation 3 is: Department of Statistics, Faculty of Sciences, Vali-e-Asr University of Rafsanjan, Rafsanjan, Iran.

The publisher apologizes for the error.
