# Supplementary material for: Classical and Bayesian estimation for type-I extended-F family with an actuarial application
Source: PLoS One. 2023 Feb 2;18(2):e0275430. doi: 10.1371/journal.pone.0275430 (PMC9894468; doi:10.1371/journal.pone.0275430)
Supplement: Attachment — Submitted filename: PONE-D-22-21984_10days.pdf [file pone.0275430.s002.pdf]

PLOS ONE

Classical and Bayesian Estimation for Type-I Extended-F Family with an Actuarial Application

--Manuscript Draft--

**Manuscript Number:**	PONE-D-22-21984
**Article Type:**	Research Article
**Full Title:**	Classical and Bayesian Estimation for Type-I Extended-F Family with an Actuarial Application
**Short Title:**	Classical and Bayesian Estimation for Type-I Extended-F Family
**Corresponding Author:**	Ahmed Z. Afify, Ph.D.<br>Benha University<br>Benha, EGYPT
**Keywords:**	Bayesian estimation; insurance data; Weibull distribution; moments; maximum likelihood; maximum product of spacings
**Abstract:**	Actuaries are often in search of heavy-tailed distributions to provide adequate fits to the insurance data. In this work, a new flexible class is proposed called type-I extended- F family. A special sub-model of the proposed class, called type-I extended-Weibull (TIEx-W) distribution is explored in detail. Basic properties of the introduced class are provided. The parameters of the TIEx-W distribution are obtained by eight classical methods of estimation and compared them using Monte Carlo simulation results for small and large samples. Besides, the Bayesian estimation of the model parameters under different loss functions for the real data set is also provided. The importance and flexibility of the TIEx-W model are illustrated by analyzing an insurance data.
**Order of Authors:**	Nada M. Alfaer, PhD<br>Sarah A. Bandar, PhD<br>Zubair Ahmad, PhD<br>Omid Kharazmi, PhD<br>Ahmed Z. Afify, Ph.D.
**Additional Information:**	
**Question**	**Response**
**Financial Disclosure**<br><br>Enter a financial disclosure statement that describes the sources of funding for the work included in this submission. Review the submission guidelines for detailed requirements. View published research articles from *PLOS ONE* for specific examples.<br><br>This statement is required for submission and **will appear in the published article** if the submission is accepted. Please make sure it is accurate.	The author(s) received no specific funding for this work.

Powered by Editorial Manager® and ProduXion Manager® from Aries Systems Corporation

**Unfunded studies**<br>Enter: *The author(s) received no specific funding for this work.*<br><br>**Funded studies**<br>Enter a statement with the following details:<br>• Initials of the authors who received each award<br>• Grant numbers awarded to each author<br>• The full name of each funder<br>• URL of each funder website<br>• Did the sponsors or funders play any role in the study design, data collection and analysis, decision to publish, or preparation of the manuscript?<br>• **NO** - Include this sentence at the end of your statement: *The funders had no role in study design, data collection and analysis, decision to publish, or preparation of the manuscript.*<br>• **YES** - Specify the role(s) played.<br><br>* typeset	
**Competing Interests**<br><br>Use the instructions below to enter a competing interest statement for this submission. On behalf of all authors, disclose any competing interests that could be perceived to bias this work—acknowledging all financial support and any other relevant financial or non-financial competing interests.<br><br>This statement is required for submission and **will appear in the published article** if the submission is accepted. Please make sure it is accurate and that any funding sources listed in your Funding Information later in the submission form are also declared in your Financial Disclosure statement.<br><br>View published research articles from *PLOS ONE* for specific examples.	The authors have declared that no competing interests exist.

**NO authors have competing interests**<br><br>Enter: *The authors have declared that no competing interests exist.*<br><br>**Authors with competing interests**<br><br>Enter competing interest details beginning with this statement:<br><br>*I have read the journal's policy and the authors of this manuscript have the following competing interests: [insert competing interests here]*<br><br>* typeset	
**Ethics Statement**<br><br>Enter an ethics statement for this submission. This statement is required if the study involved:<br><br>• Human participants<br>• Human specimens or tissue<br>• Vertebrate animals or cephalopods<br>• Vertebrate embryos or tissues<br>• Field research<br><br>Write "N/A" if the submission does not require an ethics statement.<br><br>General guidance is provided below. Consult the submission guidelines for detailed instructions. **Make sure that all information entered here is included in the Methods section of the manuscript.**	N/A

Powered by Editorial Manager® and ProduXion Manager® from Aries Systems Corporation

**Format for specific study types**<br><br>**Human Subject Research (involving human participants and/or tissue)**<br>• Give the name of the institutional review board or ethics committee that approved the study<br>• Include the approval number and/or a statement indicating approval of this research<br>• Indicate the form of consent obtained (written/oral) or the reason that consent was not obtained (e.g. the data were analyzed anonymously)<br><br>**Animal Research (involving vertebrate animals, embryos or tissues)**<br>• Provide the name of the Institutional Animal Care and Use Committee (IACUC) or other relevant ethics board that reviewed the study protocol, and indicate whether they approved this research or granted a formal waiver of ethical approval<br>• Include an approval number if one was obtained<br>• If the study involved *non-human primates*, add *additional details* about animal welfare and steps taken to ameliorate suffering<br>• If anesthesia, euthanasia, or any kind of animal sacrifice is part of the study, include briefly which substances and/or methods were applied<br><br>**Field Research**<br><br>Include the following details if this study involves the collection of plant, animal, or other materials from a natural setting:<br>• Field permit number<br>• Name of the institution or relevant body that granted permission	
**Data Availability**<br><br>Authors are required to make all data underlying the findings described fully available, without restriction, and from the time of publication. PLOS allows rare exceptions to address legal and ethical concerns. See the PLOS Data Policy and FAQ for detailed information.	Yes - all data are fully available without restriction

A Data Availability Statement describing where the data can be found is required at submission. Your answers to this question constitute the Data Availability Statement and **will be published in the article**, if accepted.<br><br>**Important:** Stating 'data available on request from the author' is not sufficient. If your data are only available upon request, select 'No' for the first question and explain your exceptional situation in the text box.<br><br>Do the authors confirm that all data underlying the findings described in their manuscript are fully available without restriction?	
**Describe where the data may be found in full sentences. If you are copying our sample text, replace any instances of XXX with the appropriate details.**<br><br>• If the data are **held or will be held in a public repository**, include URLs, accession numbers or DOIs. If this information will only be available after acceptance, indicate this by ticking the box below. For example: *All XXX files are available from the XXX database (accession number(s) XXX, XXX.).*<br>• If the data are all contained **within the manuscript and/or Supporting Information files**, enter the following: *All relevant data are within the manuscript and its Supporting Information files.*<br>• If neither of these applies but you are able to provide **details of access elsewhere**, with or without limitations, please do so. For example:<br><br>*Data cannot be shared publicly because of [XXX]. Data are available from the XXX Institutional Data Access / Ethics Committee (contact via XXX) for researchers who meet the criteria for access to confidential data.*<br><br>*The data underlying the results presented in the study are available from (include the name of the third party*	The links of the data are available in the manuscript.

and contact information or URL).<br>• This text is appropriate if the data are owned by a third party and authors do not have permission to share the data.<br><br>* typeset	
Additional data availability information:	

Powered by Editorial Manager® and ProduXion Manager® from Aries Systems Corporation

Manuscript

Click here to access/download;Manuscript;TIEx-G_plos-one.pdf

Classical and Bayesian Estimation for Type-I Extended-F Family with an Actuarial Application

Nada M. Alfaer[1◑], Sarah A. Bandar [2◑], Zubair Ahmad [3◑], Omid Kharazmi[4◑], Ahmed Z. Afify [5◑]

1 Department of Mathematics & Statistics, College of Science, Taif University, P.O. Box 11099, Taif 21944, Saudi Arabia
2 Department of Mathematics, College of Education, Misan University, Amarah, Iraq
3 Department of Statistics, Yazd University, P.O. Box 89175-741, Yazd, Iran
4 Department of Statistics, Faculty of Sciences, Vali-e-Asr University of Rafsanjan, Rafsanjan, Iran
5 Department of Statistics, Mathematics and Insurance, Benha University, Benha 13511, Egypt

◑These authors contributed equally to this work.
‡These authors also contributed equally to this work.
* ahmed.afify@fcom.bu.edu.eg

Abstract

Actuaries are often in search of heavy-tailed distributions to provide adequate fits to the insurance data. In this work, a new flexible class is proposed called type-I extended-F family. A special sub-model of the proposed class, called type-I extended-Weibull (TIEx-W) distribution is explored in detail. Basic properties of the introduced class are provided. The parameters of the TIEx-W distribution are obtained by eight classical methods of estimation and compared them using Monte Carlo simulation results for small and large samples. Besides, the Bayesian estimation of the model parameters under different loss functions for the real data set is also provided. The importance and flexibility of the TIEx-W model are illustrated by analyzing an insurance data.

Keywords: Bayesian estimation; insurance data; Weibull distribution; moments; maximum likelihood; maximum product of spacings.

1 Introduction

Modeling insurance losses data using heavy-tailed distributions is an important subject matter for risk managers and actuaries. Generally, the insurance sets of data are usually right-skewed, hump-shaped, unimodal, and have thick right tail. Distributions possessing such characteristics are considered prominent candidates for modeling such heavy-tailed data. For more details the interested reader can see Resnick (1997) and McNeil (1997). The heavy-tailed distributions are adopted to estimate insurance losses data and thereby helps in assessing of business risk level. Hence, due to its immense significance in actuarial sciences, these types of data are studied and explored extensively and several distributions are introduced in the actuarial literature.

The insurance loss data, financial returns, file sizes on network servers etc, are explored and modeled by several models such as the Lomax (Scollnik, 2007), Pareto (Coorey and Ananda, 2008), Weibull (Scollnik and Sun, 2012), Burr (Nadarajah and

Bakar, 2014), log-logistic (Bakar et al., 2015), Stoppa (Calderin et al., 2016) distributions, among others.

However, the classical distributions are still not flexible enough to adequately fit such data sets, and some distributions do not possess closed form for its cumulative distribution function (cdf) causing difficulties related to the parameter estimation. For example, (i) the Pareto distribution has a monotonically decreasing shape for its probability density function (pdf), and it does not often provide best fit to many data sets, (ii) Weibull distribution is suitable for modeling small losses, but, unfortunately, it is not suitable for modeling large losses, and (iii) log-normal distribution does not have closed form solution of cdf result in estimation consequences.

To overcome the aforementioned problems, the researchers have been working on to propose new distributions to address these issues. For example, the heavy-tailed distributions by Beirlant et al. (2001), Pareto–Levy by Coronel-Brizio et al. (2005), loss models by Klugman et al. (2012), Weibull-Pareto by Scollnik and Sun (2012), generalized log-Moyal by Bhati and Ravi (2018), and Generalized Pareto by Ghitany et al. (2018).

In the recent era, the statisticians have shown an increased interest to propose new family of distributions by introducing additional parameters. In this credit, Mahdavi and Kundu (2017) pioneered a new method, called the alpha-power transformation (APT) family, for generating univariate distributions using the following cdf

$$G\left(x;\alpha,\xi\right)=\frac{\alpha^{F(x;\xi)}-1}{\alpha-1},\qquad \alpha,\xi>0,\alpha\neq 1,x\in\mathbb{R}. \tag{1}$$

The AP-F class is studied with more detail in Mead et al. (2019). Ahmad et al. (2019) proposed another approach, called extended APT (Ex-APT) family for generating new distributions which is specified by the cdf

$$G\left(x;\alpha,\xi\right)=\frac{\alpha^{F(x;\xi)}-e^{F(x;\xi)}}{\alpha-e},\qquad \alpha,\xi>0,\alpha\neq 1,\alpha>e,x\in\mathbb{R}. \tag{2}$$

Ampadu (2019) introduced another new method called, Ampadu APT family which is defined by the cdf

$$G\left(x;\alpha,\xi\right)=\frac{e^{-F(x;\xi)}-\alpha^{F(x;\xi)}}{e^{-1}-\alpha},\quad \xi>0,\ \alpha>\frac{1}{e},\ x\in\mathbb{R}. \tag{3}$$

In this work, we address a new class to generate new flexible distributions, called type-I extended-F (TIEx-F) family which has the following cdf

$$G\left(x;\eta,\xi\right)=\frac{e^{F(x;\xi)}-\left[1-\eta F\left(x;\xi\right)\right]}{e-\bar{\eta}},\quad \eta,\xi>0,\eta\neq 1-e,x\in\mathbb{R}. \tag{4}$$

where $F\left(x;\xi\right)$ is cdf of the baseline random variable (rv) depending on the vector of parameters ξ and η is an additional parameter.

The pdf corresponding to (4) takes the form

$$g\left(x;\eta,\xi\right)=\frac{f\left(x;\xi\right)\left[e^{F(x;\xi)}+\eta\right]}{e-\bar{\eta}},\quad x\in\mathbb{R}. \tag{5}$$

The new pdf 5 of the TIEx-F family will be most tractable for simple analytical expressions of $F\left(x;\xi\right)$ and $f\left(x;\xi\right)$. Henceforth, a rv X with pdf (5)is denoted by $X\sim$ TIEx-$F(\eta,\xi)$. The TIEx-F family has some desirable characteristics such as, it is novel and very simple approach of adding an additional parameter to generalize the existing distributions, hence it can provide extended versions of baseline distributions with closed form expressions for their cdf and hazard rate function (hrf). The TIEx-F family

provides better fit than other competing modified models under the same baseline model. Further a new lifetime model, based on the APTTIEx-F family, called the TIEx-Weibull (TIEx-W) distribution is studied.

Additionally, the TIEx-W parameters are estimated by using several estimation methods and their performance is explored by detailed simulations for small and large samples. Many authors have addressed different estimators to estimate the parameters of generalized models such as the Weibull–Marshall–Olkin power-Lindley Al-Babtain et al. (2021), Marshall–Olkin–Weibull exponential Afify et al. (2022) distributions.

The rest of this work is unfolded as follows. In Section 2, we present a special sub-model of the proposed method. Some characteristics are provided in Section 3. Section 4 is devoted to the estimation of the model parameters with a detailed simulation study. A real data application is discussed in Section 5. Bayesian estimation under five loss functions are explored in Section 6. The paper is concluded in Section 7.

2 The TIEx-W Distribution

Using the cdf of the Weibull (W) distribution, $F(x) = 1 - e^{-\gamma x^{\alpha}}, \ \ , x > 0, \ \gamma, \ \alpha > 0$ and its pdf, $f(x) = \alpha\gamma x^{\alpha-1} e^{-\gamma x^{\alpha}}$, we obtain the cdf of the TIEx-W distribution

$$G(x) = \frac{e^{\left(1-e^{-\gamma x^{\alpha}}\right)} - \left[1 - \eta\left(1 - e^{-\gamma x^{\alpha}}\right)\right]}{e - \bar{\eta}}, \qquad \alpha, \eta, \gamma > 0, x > 0. \tag{6}$$

The corresponding pdf of the TIEx-W distribution reduces to

$$g(x) = \frac{\alpha\gamma x^{\alpha-1} e^{-\gamma x^{\alpha}} \left(e^{\left(1-e^{-\gamma x^{\alpha}}\right)} + \eta\right)}{e - \bar{\eta}}, \qquad x > 0. \tag{7}$$

Figure 1 sketches some density plots for the TIEx-W distribution.

3 Quantile Function and Moments

In this section, we derive some general properties of the TIEx-F family including quantile function and moments.

The quantile function (qf) of the TIEx-F class follows by inverting the cdf (4). Thus, we have

$$Q(u) = G^{-1}(u) = F^{-1}(t), \tag{8}$$

where t represents the solution of $e^{t} - 1 + \eta t - u(e - \bar{\eta}) = 0$, and u follows the uniform distribution (0, 1). Equation (8) can be adopted to generate random numbers from the TIEx-F distributions.

Let $X \sim$ TIEx-$F(\eta, \xi)$, hence the rth moment of X takes the form

$$\mu'_r = \int_{-\infty}^{\infty} x^r g(x; \eta, \xi)\, dx. \tag{9}$$

By inserting (5) in (9), we obtain

$$\mu'_r = \int_{-\infty}^{\infty} x^r \frac{f(x,\xi)\left[e^{F(x,\xi)} + \eta\right]}{(e - \bar{\eta})} dx. \tag{10}$$

The Maclaurin series is applied to the exponential function e^x as follows

$$e^x = 1 + x + \frac{x^2}{2!} + \frac{x^3}{3!} + ... = \sum_{n=0}^{\infty} \frac{x^n}{n!}. \tag{11}$$

Using $x = F(x;\xi)$ in (11)

$$e^{F(x;\xi)} = \sum_{n=0}^{\infty} \frac{F(x;\xi)^n}{n!}, \tag{12}$$

Hence, the rth moment follows as

$$\mu_r' = \sum_{n=0}^{\infty} \frac{\Lambda_{r,n}}{(e-\bar{\eta})\, n!} + \frac{\eta \Lambda_r}{(e-\bar{\eta})}, \tag{13}$$

where $\Lambda_{r,n} = \int\limits_{-\infty}^{\infty} x^r \; f(x,\xi) \;\; F(x;\xi)^n \, dx$, and $\Lambda_n = \int\limits_{-\infty}^{\infty} x^r \; f(x,\xi) \; dx$.

The moment generating function of the TIEx-F family has the form $M_x(t)$, is given by

$$M_x(t) = \sum_{r,n=0}^{\infty} \frac{t^r \Lambda_{r,n}}{(e-\bar{\eta})\, r! n!} + \sum_{r=0}^{\infty} \frac{t^r \eta \Lambda_r}{(e-\bar{\eta})\, r!}.$$

The first four moments of the TIEx-F family follows for r =1,2,3,4. Numerical values for the mean, variance, skewness (Sk) and kurtosis (Kur) of the TIEx-W distribution for some parametric values are reported in Tables **??** and **??**. These tables show that the new additional parameter provides more flexibility to the TIEx-W distribution in terms of its Sk and Kur.

4 Estimation for the TIEx-W Parameters

In this section, eight estimation methods are considered to estimate the unknown parameters of the TIEx-W model.

4.1 Maximum Likelihood Method

Consider a random sample from the TIEx-W model with pdf given by (7), denoted by $x_1, ..., x_m$, and their associated observed order statistics, denoted by $x_{(1)}, x_{(2)}, \cdots, x_{(n)}$. Then, the log-likelihood function reduces to

$$\begin{aligned} L(\alpha,\gamma,\eta) =& m\log(\alpha\,\gamma) - m\log(e-\bar{\eta}) - (\alpha-1)\sum_{k=1}^{m}\log(x_k) \\ & - \gamma\sum_{k=1}^{m} x_k^{\alpha} + \sum_{k=1}^{m}\log\left(e^{\left(1-e^{-\gamma x_k^{\alpha}}\right)} + \eta\right). \end{aligned} \tag{14}$$

The maximum likelihood estimates (MLEs) of α, γ and η can be determined by maximizing (14) with respect to α, γ and η or by solving the following two non linear equations

$$\frac{\partial L(\alpha,\gamma,\eta)}{\partial\alpha} = \frac{m}{\alpha} - \sum_{k=1}^{m}\log(x_k) - \alpha\,\gamma\sum_{k=1}^{m} x_k^{\alpha-1} + \sum_{k=1}^{m} \frac{\gamma x_k^{\alpha} e^{\left(1-e^{-\gamma x_k^{\alpha}}\right)}\log(x_k)}{e^{\gamma x_k^{\alpha}}\left(e^{\left(1-e^{-\gamma x_k^{\alpha}}\right)} + \eta\right)} = 0, \tag{15}$$

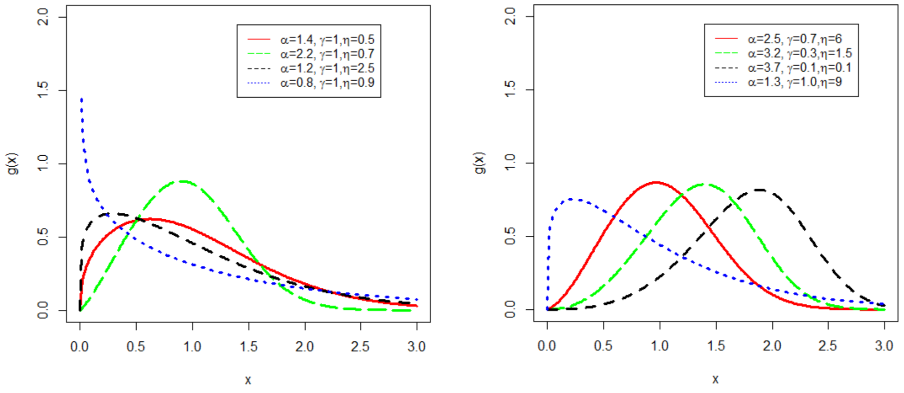

Fig 1. Some density plots for the TIEx-W distribution.

Table 1. Descriptive measures of TIEx-W distribution for $\alpha = 1.5$, $\gamma = 1$ and several values of η.

η	Mean	Variance	Sk	Kur
0.5	1.034349	0.415879	0.886898	3.900027
3	0.964618	0.398899	0.978853	4.113748
6	0.940569	0.390788	1.014008	4.211114
9	0.929982	0.386850	1.029937	4.258030
12	0.914026	0.324536	1.139009	4.485534
15	0.900207	0.293016	1.494864	5.30359
18	0.855505	0.255408	1.678955	5.816348

Table 2. Descriptive measures of TIEx-W distribution for $\eta = 1.5$, $\gamma = 1$ and several values of α.

α	Mean	Variance	Sk	Kur
0.9	1.226517	1.605750	2.106404	9.899798
1.5	1.002782	0.409397	0.926495	3.984561
3	0.946664	0.106607	0.059345	2.708730
6	0.956762	0.031269	-0.47328	3.175183
12	0.966997	0.015069	-0.68961	3.660633
15	0.973592	0.008880	-0.80966	4.012897
18	0.978053	0.005856	-0.88636	4.266952

$$\frac{\partial L(\alpha,\gamma,\eta)}{\partial\gamma} = \frac{m}{\gamma} - \sum_{k=1}^{m} x_k^{\alpha} + \sum_{k=1}^{m} \frac{x_k^{\alpha} e^{-\gamma x_k^{\alpha}} e^{\left(1-e^{-\gamma x_k^{\alpha}}\right)}}{e^{\left(1-e^{-\gamma x_k^{\alpha}}\right)} + \eta} = 0 \tag{16}$$

and

$$\frac{\partial L(\alpha,\gamma,\eta)}{\partial\eta} = \frac{m}{(\bar{\eta} - e)} + \sum_{k=1}^{m} \frac{1}{e^{\left(1-e^{-\gamma x_k^{\alpha}}\right)} + \eta} = 0. \tag{17}$$

The three Equations (15 - 17) have no explicit solutions, hence numerical techniques will be employed to obtain the MLEs of the parameters.

4.2 Maximum Product of Spacings Method

The maximum product of spacings estimates (MPSEs) for α, γ and η of the TIEx-W model can be obtained by maximizing the following function with respect to α, γ and η

$$\begin{aligned} M(\alpha,\gamma,\eta) = \frac{1}{m+1} \sum_{k=1}^{m+1} \log \Bigg\{ & \frac{e^{\left(1-e^{-\gamma x_k^{\alpha}}\right)} - \left[1 - \eta\left(1 - e^{-\gamma x_k^{\alpha}}\right)\right]}{e - \bar{\eta}} \\ & - \frac{e^{\left(1-e^{-\gamma x_{k-1}^{\alpha}}\right)} - \left[1 - \eta\left(1 - e^{-\gamma x_{k-1}^{\alpha}}\right)\right]}{e - \bar{\eta}} \Bigg\}. \end{aligned} \tag{18}$$

The MPSEs of the parameters α, γ and η, denoted by $\hat{\alpha}_{MP}$, $\hat{\gamma}_{MP}$ and $\hat{\eta}_{MP}$, follows by maximizing Equation (18) or by solving the following three equations simultaneously

$$\frac{\partial M(\alpha,\gamma,\eta)}{\partial\alpha} = \frac{1}{m+1} \sum_{k=1}^{m+1} \frac{\varrho_k(\alpha,\gamma,\eta) - \varrho_{k-1}(\alpha,\gamma,\eta)}{\upsilon_k(\alpha,\gamma,\eta)} = 0,$$

$$\frac{\partial M(\alpha,\gamma,\eta)}{\partial\gamma} = \frac{1}{m+1} \sum_{k=1}^{m+1} \frac{\vartheta_k(\alpha,\gamma,\eta) - \vartheta_{k-1}(\alpha,\gamma,\eta)}{\upsilon_k(\alpha,\gamma,\eta)} = 0$$

and

$$\frac{\partial M(\alpha,\gamma,\eta)}{\partial\eta} = \frac{1}{m+1} \sum_{k=1}^{m+1} \frac{\varphi_k(\alpha,\gamma,\eta) - \varphi_{k-1}(\alpha,\gamma,\eta)}{\upsilon_k(\alpha,\gamma,\eta)} = 0,$$

where

$$\begin{aligned} \upsilon_k(\alpha,\gamma,\eta) = \Bigg\{ & \frac{e^{\left(1-e^{-\gamma x_k^{\alpha}}\right)} - \left[1 - \eta\left(1 - e^{-\gamma x_k^{\alpha}}\right)\right]}{e - \bar{\eta}} \\ & - \frac{e^{\left(1-e^{-\gamma x_{k-1}^{\alpha}}\right)} - \left[1 - \eta\left(1 - e^{-\gamma x_{k-1}^{\alpha}}\right)\right]}{e - \bar{\eta}} \Bigg\}, \end{aligned}$$

$$\varrho_k(\alpha,\gamma,\eta) = \frac{1}{(e - \bar{\eta})} \gamma x_k^{\alpha} e^{-\gamma x_k^{\alpha}} \left[\eta + e^{\left(1-e^{-\gamma x_k^{\alpha}}\right)}\right] \log(x_k), \tag{19}$$

$$\vartheta_k(\alpha,\gamma,\eta) = \frac{1}{(e - \bar{\eta})} x_k^{\alpha} e^{-\gamma x_k^{\alpha}} \left[\eta + e^{\left(1-e^{-\gamma x_k^{\alpha}}\right)}\right] \tag{20}$$

and

$$\varphi_k(\alpha,\gamma,\eta)=\frac{1}{(e-\bar{\eta})^4}\left\{(e-\bar{\eta})^3\left(1-e^{-\gamma x_k^\alpha}\right)-\left[1-\eta\left(1-e^{-\gamma x_k^\alpha}\right)\right]\right\}-\frac{e^{1-e^{-\gamma x_k^\alpha}}}{(e-\bar{\eta})^2}. \tag{21}$$

4.3 Least Squares and Weighted Least Squares Methods

The least squares estimates (LSEs) and weighted least squares estimates (WLSEs) Swain (1988) of the TIEx-W parameters can be obtained by minimizing the following function

$$S(\alpha,\gamma,\eta) \;=\; \sum_{k=1}^{m}\delta_k\left\{\frac{e^{\left(1-e^{-\gamma x_k^\alpha}\right)}-\left[1-\eta\left(1-e^{-\gamma x_k^\alpha}\right)\right]}{e-\bar{\eta}}-\frac{k}{m+1}\right\}^2,$$

where $\delta_k=\frac{(m+2)\,(m+1)^2}{k(m-k+1)}$ for the WLS method and $\delta_k=1$ for the LS method. Practically, the LSEs of the parameters α, γ and η of the TIEx-W model, denoted by $\hat{\alpha}_{LS}$, $\hat{\gamma}_{Ls}$ and $\hat{\eta}_{LS}$, and the WLSEs, denoted by $\hat{\alpha}_{WL}$, $\hat{\gamma}_{WL}$ and $\hat{\eta}_{WL}$, are obtained by solving the following two equations simultaneously

$$\frac{\partial S(\alpha,\gamma,\eta)}{\partial\alpha} \;=\; \sum_{k=1}^{m}\delta_k\left\{\frac{e^{\left(1-e^{-\gamma x_k^\alpha}\right)}-\left[1-\eta\left(1-e^{-\gamma x_k^\alpha}\right)\right]}{e-\bar{\eta}}-\frac{k}{m+1}\right\}\varrho_k(\alpha,\gamma,\eta)=0,$$

$$\frac{\partial S(\alpha,\gamma,\eta)}{\partial\gamma}=\sum_{k=1}^{m}\delta_k\left\{\frac{e^{\left(1-e^{-\gamma x_k^\alpha}\right)}-\left[1-\eta\left(1-e^{-\gamma x_k^\alpha}\right)\right]}{e-\bar{\eta}}-\frac{k}{m+1}\right\}\vartheta_k(\alpha,\gamma,\eta)=0$$

and

$$\frac{\partial S(\alpha,\gamma,\eta)}{\partial\eta}=\sum_{k=1}^{m}\delta_k\left\{\frac{e^{\left(1-e^{-\gamma x_k^\alpha}\right)}-\left[1-\eta\left(1-e^{-\gamma x_k^\alpha}\right)\right]}{e-\bar{\eta}}-\frac{k}{m+1}\right\}\varphi_k(\alpha,\gamma,\eta)=0,$$

where $\varrho_k(\alpha,\gamma,\eta)$, $\vartheta_k(\alpha,\gamma,\eta)$ and $\varphi_k(\alpha,\gamma,\eta)$ are given by (19)-(21).

4.4 Cramér-von-Mises and Percentiles Methods

The Cramér-von Mises estimates (CVMEs) of the parameters of the TIEx-W model, say $\hat{\alpha}_{CM}$ and $\hat{\gamma}_{CM}$, follows by minimizing the following equation with respect to the parameters α, γ and η

$$C(\alpha,\gamma,\eta) \;=\; \sum_{k=1}^{m}\left\{\frac{e^{\left(1-e^{-\gamma x_k^\alpha}\right)}-\left[1-\eta\left(1-e^{-\gamma x_k^\alpha}\right)\right]}{e-\bar{\eta}}-\frac{2k-1}{2m}\right\}^2. \tag{22}$$

Equivalently, the CVMEs of the parameters α, γ and η are obtained by solving the following two equations

$$\frac{\partial C(\alpha,\gamma,\eta)}{\partial\alpha} \;=\; \sum_{k=1}^{m}\left\{\frac{e^{\left(1-e^{-\gamma x_k^\alpha}\right)}-\left[1-\eta\left(1-e^{-\gamma x_k^\alpha}\right)\right]}{e-\bar{\eta}}-\frac{2k-1}{2m}\right\}\varrho_k(\alpha,\gamma,\eta)=0,$$

$$\frac{\partial C(\alpha,\gamma,\eta)}{\partial\gamma}=\sum_{k=1}^{m}\left\{\frac{e^{\left(1-e^{-\gamma x_k^{\alpha}}\right)}-\left[1-\eta\left(1-e^{-\gamma x_k^{\alpha}}\right)\right]}{e-\bar{\eta}}-\frac{2k-1}{2m}\right\}\vartheta_k(\alpha,\gamma,\eta)=0$$

and

$$\frac{\partial C(\alpha,\gamma,\eta)}{\partial\eta}=\sum_{k=1}^{m}\left\{\frac{e^{\left(1-e^{-\gamma x_k^{\alpha}}\right)}-\left[1-\eta\left(1-e^{-\gamma x_k^{\alpha}}\right)\right]}{e-\bar{\eta}}-\frac{2k-1}{2m}\right\}\varphi_k(\alpha,\gamma,\eta)=0,$$

$\varrho_k(\alpha,\gamma,\eta)$, $\vartheta_k(\alpha,\gamma,\eta)$ and $\varphi_k(\alpha,\gamma,\eta)$ are given by (19)-(21).

The percentiles estimation method was proposed by Kao (1958, 1959). The percentiles estimates (PCEs) of the TIEx-W parameters α, γ and η, denoted by $\hat{\alpha}_{PE}$, $\hat{\gamma}_{PE}$ and $\hat{\eta}_{PE}$, can be obtained by minimizing

$$P(\alpha,\gamma,\eta) \quad = \quad \sum_{k=1}^{m}\left\{x_{(k)}-Q(u)\right\}^2, \tag{23}$$

where $Q(u)$ denotes the quantile function of the TIEx-X distribution and it has no closed form expression. Then, numerical techniques are employed to generate data from the TIEx-X distribution.

4.5 Anderson-Darling and Right-Tail Anderson-Darling Methods

The Anderson-Darling (AD) method is known as a type of minimum distance estimators which can be obtained by minimizing the AD statistic. For the TIEx-W model, the AD estimates (ADEs) of the TIEx-W parameters α, γ and η, say $\hat{\alpha}_{AD}$, $\hat{\gamma}_{AD}$ and $\hat{\eta}_{AD}$, can be obtained by minimizing

$$AD(\alpha,\gamma,\eta) \quad = \quad -m-\frac{1}{m}\sum_{k=1}^{m}(2k-1)[\log(F(x_{(k)}|\alpha,\gamma,\eta))+\log(\bar{F}(x_{(m-k+1)}|\alpha,\gamma,\eta))],$$

with respect to α, γ and η, where $\bar{F}(.)=1-F(.)$. These estimates can also be obtained by solving the following equations

$$\frac{\partial AD(\alpha,\gamma,\eta)}{\partial\alpha}=\sum_{k=1}^{m}(2k-1)\frac{\varrho_k(\alpha,\gamma,\eta)}{F(x_{(k)}|\alpha,\gamma,\eta)}-\sum_{k=1}^{m}(2k-1)\frac{\varrho_{m-k+1}(\alpha,\gamma,\eta)}{1-F(x_{(m-k+1)}|\alpha,\gamma,\eta)}=0,$$

$$\frac{\partial AD(\alpha,\gamma,\eta)}{\partial\gamma}=\sum_{k=1}^{m}(2k-1)\frac{\vartheta_k(\alpha,\gamma,\eta)}{F(x_{(k)}|\alpha,\gamma,\eta)}-\sum_{k=1}^{m}(2k-1)\frac{\vartheta_{m-k+1}(\alpha,\gamma,\eta)}{1-F(x_{(m-k+1)}|\alpha,\gamma,\eta)}=0$$

and

$$\frac{\partial AD(\alpha,\gamma,\eta)}{\partial\gamma}=\sum_{k=1}^{m}(2k-1)\frac{\varphi_k(\alpha,\gamma,\eta)}{F(x_{(k)}|\alpha,\gamma,\eta)}-\sum_{k=1}^{m}(2k-1)\frac{\varphi_{m-k+1}(\alpha,\gamma,\eta)}{1-F(x_{(m-k+1)}|\alpha,\gamma,\eta)}=0,$$

where $\varrho_k(\alpha,\gamma,\eta)$, $\vartheta_k(\alpha,\gamma,\eta)$ and $\varphi_k(\alpha,\gamma,\eta)$ are given by (19)-(21).

Similarly, the right-tail Anderson-Darling estimates (RADEs) of the TIEx-W parameters α, γ and η, say $\hat{\alpha}_{RAD}$, $\hat{\gamma}_{RAD}$ and $\hat{\eta}_{RAD}$, are obtained by minimizing

$$RAD(\alpha,\gamma,\eta) = \frac{m}{2} - 2\sum_{k=1}^{n} F\left(x_{(k)} \mid \alpha,\gamma,\eta\right) - \frac{1}{m}\sum_{k=1}^{n}(2k-1)\log \overline{F}\left(x_{(m-k+1)} \mid \alpha,\gamma,\eta\right),$$

with respect to α, γ and η. Furthermore, these estimates can also be obtained by solving the following equations simultaneously

$$\frac{\partial RAD(\alpha,\gamma,\eta)}{\partial\alpha} = -2\sum_{k=1}^{m}\varrho_k(\alpha,\gamma,\eta) + \frac{1}{m}\sum_{k=1}^{m}(2k-1)\frac{\varrho_{m-k+1}(\alpha,\gamma,\eta)}{1-F(x_{(m-k+1)}|\alpha,\gamma,\eta)} = 0,$$

$$\frac{\partial RAD(\alpha,\gamma,\eta)}{\partial\gamma} = -2\sum_{k=1}^{m}\vartheta_k(\alpha,\gamma,\eta) + \frac{1}{m}\sum_{k=1}^{m}(2k-1)\frac{\vartheta_{m-k+1}(\alpha,\gamma,\eta)}{1-F(x_{(m-k+1)}|\alpha,\gamma,\eta)} = 0$$

and

$$\frac{\partial RAD(\alpha,\gamma,\eta)}{\partial\eta} = -2\sum_{k=1}^{m}\varphi_k(\alpha,\gamma,\eta) + \frac{1}{m}\sum_{k=1}^{m}(2k-1)\frac{\varphi_{m-k+1}(\alpha,\gamma,\eta)}{1-F(x_{(m-k+1)}|\alpha,\gamma,\eta)} = 0,$$

where $\varrho_k(\alpha,\gamma,\eta)$, $\vartheta_k(\alpha,\gamma,\eta)$ and $\varphi_k(\alpha,\gamma,\eta)$ are given by (19)-(21).

5 Simulation Results

In this section, we have carried out an extensive simulation study to assess and compare the performance of the eight frequentist estimators. The methods are explored for $n = \{20, 50, 100, 200, 400\}$ with parameter values $\alpha = (2.75)$, $\gamma = (0.5, 2.0)$ and $\eta = (0.67, 1.5)$. We generate $N = 5,000$ random samples from the TIEx-W distribution using the inverse transform method. The following procedures are adopted to generate the data from the TIEx-W distribution.

- Generate random values from the TIEx-W distribution with size n.
- Using the obtained samples in step 1, calculate $\widehat{\alpha}$, $\widehat{\gamma}$ and $\widehat{\eta}$ via 1-MLES, 2-MPSEs, 3-LSEs, 4-CVMEs, 5-WLSEs, 6-PCEs, 7-ADEs, 8-RADEs.
- Repeat the steps 1 and 2, N times.

For each estimate, we calculate average estimates of the parameters (AVEs), mean-squared errors (MSEs), absolute bias (ABs), and mean relative errors (MREs). The formulae of these measures are given for $\boldsymbol{\phi} = (\alpha,\gamma,\eta)^{\intercal}$ by

$MSEs = \frac{1}{N}\sum_{i=1}^{N}(\widehat{\boldsymbol{\phi}} - \boldsymbol{\phi})^2$, ABs, $|ABs(\widehat{\boldsymbol{\phi}})| = \frac{1}{N}\sum_{i=1}^{N}|\widehat{\boldsymbol{\phi}} - \boldsymbol{\phi}|$, and MREs, $MREs = \frac{1}{N}\sum_{i=1}^{N}|\widehat{\boldsymbol{\phi}} - \boldsymbol{\phi}|/\boldsymbol{\phi}$.

The performance of the considered estimators are evaluated in terms of absolute bias, mean-squared error and mean relative error. Considering this approach, the most efficient estimation method will be the one whose MREs value is closer to one and bias closer to zero. All simulations are conducted via R software.

In Tables 3-5 we report the values of AVEs, MSEs, ABs, and MREs for the WLSEs, LSEs, MLEs, MPSEs, CVMEs, ADEs, RADEs and PCEs. The results show that all estimators reveal the property of consistency, where the MSEs and MREs decrease as sample size increases, for all parameter combinations. All estimators show the property of consistency for all parameter combinations. In summary, we conclude that the maximum likelihood (ML) method outperforms all other estimation methods. Therefore, ML methods is considered the optimal method for estimating the TIEx-W parameters.

Table 3. Simulation results of the eight estimation methods for $\varphi = (\alpha = 2.75, \gamma = 2.00, \eta = 0.67)^\intercal$

n	Measures	Est. Par.	MLEs	MPSEs	LSEs	CVMEs	WLSEs	PCEs	ADEs	RADEs
20	AVEs	$\hat{\alpha}$	2.75624	2.35767	2.39565	2.64250	2.40206	2.42096	2.62632	2.77081
		$\hat{\gamma}$	1.72155	2.19706	1.99302	1.72319	2.24302	2.05615	1.89511	2.16619
		$\hat{\eta}$	0.74185	0.59915	0.64737	0.73176	0.59813	0.63529	0.69099	0.64290
	MSEs	$\hat{\alpha}$	1.44036	1.39733	2.13957	2.34009	1.41448	1.79126	1.46813	2.40870
		$\hat{\gamma}$	2.85766	3.17883	3.82296	3.73225	3.31452	3.60669	3.10305	3.68172
		$\hat{\eta}$	0.21730	0.22421	0.34959	0.33569	0.26117	0.32641	0.25616	0.32126
	ABs	$\hat{\alpha}$	1.20015	1.18209	1.46273	1.52974	1.18932	1.33838	1.21166	1.55200
		$\hat{\gamma}$	1.69046	1.78293	1.95524	1.93190	1.82058	1.89913	1.76155	1.91878
		$\hat{\eta}$	0.46616	0.47351	0.59126	0.57939	0.51105	0.57132	0.50612	0.56680
	MREs	$\hat{\alpha}$	0.43642	0.42985	0.53190	0.55627	0.43248	0.48668	0.44060	0.56436
		$\hat{\gamma}$	0.84523	0.89146	0.97762	0.96595	0.91029	0.94956	0.88077	0.95939
		$\hat{\eta}$	0.69576	0.70673	0.88248	0.86476	0.76275	0.85272	0.75540	0.84597
50	AVEs	$\hat{\alpha}$	2.76195	2.51121	2.58904	2.79208	2.68955	2.61937	2.72290	2.79844
		$\hat{\gamma}$	1.91971	2.12357	1.92990	2.12100	2.27972	1.94300	1.96959	2.17193
		$\hat{\eta}$	0.69372	0.62512	0.67348	0.65466	0.60808	0.67164	0.67847	0.64505
	MSEs	$\hat{\alpha}$	0.57487	0.55864	1.07358	1.12896	0.64893	0.77387	0.66492	1.21394
		$\hat{\gamma}$	1.48154	1.54704	2.57226	2.53167	1.89094	1.93310	1.67940	2.33034
		$\hat{\eta}$	0.07457	0.07332	0.16618	0.16265	0.09270	0.10313	0.08899	0.11982
	ABs	$\hat{\alpha}$	0.75820	0.74742	1.03614	1.06253	0.80556	0.87970	0.81543	1.10179
		$\hat{\gamma}$	1.21719	1.24380	1.60383	1.59112	1.37512	1.39036	1.29592	1.52654
		$\hat{\eta}$	0.27307	0.27077	0.40766	0.40330	0.30447	0.32114	0.29830	0.34616
	MREs	$\hat{\alpha}$	0.27571	0.27179	0.37678	0.38637	0.29293	0.31989	0.29652	0.40065
		$\hat{\gamma}$	0.60859	0.62190	0.80191	0.79556	0.68756	0.69518	0.64796	0.76327
		$\hat{\eta}$	0.40756	0.40414	0.60844	0.60194	0.45443	0.47932	0.44523	0.51665
100	AVEs	$\hat{\alpha}$	2.75987	2.62363	2.70171	2.70120	2.76422	2.68455	2.73279	2.81653
		$\hat{\gamma}$	1.98141	2.08244	2.07212	1.94446	2.27257	1.95607	1.98239	2.11577
		$\hat{\eta}$	0.67592	0.63876	0.65424	0.68058	0.61564	0.67465	0.67247	0.65215
	MSEs	$\hat{\alpha}$	0.26759	0.25780	0.58047	0.54830	0.32075	0.37360	0.35242	0.57912
		$\hat{\gamma}$	0.75494	0.79775	1.60433	1.52309	1.03080	1.10899	0.94792	1.35441
		$\hat{\eta}$	0.03084	0.03008	0.07602	0.07596	0.04202	0.05023	0.04228	0.05593
	ABs	$\hat{\alpha}$	0.51729	0.50774	0.76189	0.74048	0.56635	0.61123	0.59365	0.76100
		$\hat{\gamma}$	0.86887	0.89317	1.26662	1.23414	1.01528	1.05308	0.97361	1.16379
		$\hat{\eta}$	0.17561	0.17344	0.27572	0.27561	0.20498	0.22413	0.20563	0.23649
	MREs	$\hat{\alpha}$	0.18811	0.18463	0.27705	0.26926	0.20594	0.22226	0.21587	0.27673
		$\hat{\gamma}$	0.43444	0.44658	0.63331	0.61707	0.50764	0.52654	0.48680	0.58190
		$\hat{\eta}$	0.26210	0.25887	0.41153	0.41136	0.30594	0.33452	0.30691	0.35297
200	AVEs	$\hat{\alpha}$	2.75079	2.66858	2.72422	2.75894	2.76600	2.71390	2.74620	2.78890
		$\hat{\gamma}$	1.96534	2.04181	1.97628	2.02185	2.15149	1.98707	2.01250	2.06069
		$\hat{\eta}$	0.67979	0.65299	0.67278	0.66696	0.63767	0.67100	0.66785	0.65997
	MSEs	$\hat{\alpha}$	0.13080	0.11065	0.29241	0.29518	0.16193	0.17075	0.17373	0.29994
		$\hat{\gamma}$	0.40931	0.34939	0.84520	0.88509	0.49341	0.52890	0.51518	0.70687
		$\hat{\eta}$	0.01637	0.01194	0.03816	0.03816	0.01861	0.02122	0.02068	0.02692
	ABs	$\hat{\alpha}$	0.36166	0.33265	0.54075	0.54331	0.40240	0.41322	0.41681	0.54767
		$\hat{\gamma}$	0.63977	0.59109	0.91935	0.94079	0.70243	0.72725	0.71776	0.84076
		$\hat{\eta}$	0.12794	0.10929	0.19534	0.19536	0.13640	0.14569	0.14380	0.16408
	MREs	$\hat{\alpha}$	0.13151	0.12096	0.19664	0.19757	0.14633	0.15026	0.15157	0.19915
		$\hat{\gamma}$	0.31989	0.29554	0.45967	0.47040	0.35122	0.36363	0.35888	0.42038
		$\hat{\eta}$	0.19096	0.16312	0.29155	0.29158	0.20359	0.21744	0.21463	0.24490
400	AVEs	$\hat{\alpha}$	2.74732	2.70582	2.73334	2.75649	2.77728	2.72239	2.76015	2.75716
		$\hat{\gamma}$	1.99043	2.01837	2.01833	2.00434	2.12210	2.00064	2.02093	2.01438
		$\hat{\eta}$	0.67117	0.65957	0.66592	0.67495	0.64610	0.67013	0.66668	0.66763
	MSEs	$\hat{\alpha}$	0.06783	0.03960	0.14594	0.13372	0.07942	0.08860	0.08245	0.15177
		$\hat{\gamma}$	0.20478	0.01276	0.50480	0.43692	0.24577	0.28979	0.28083	0.37956
		$\hat{\eta}$	0.00753	0.00173	0.01906	0.01747	0.00928	0.01104	0.01091	0.01383
	ABs	$\hat{\alpha}$	0.26044	0.19900	0.38202	0.36568	0.28182	0.29765	0.28715	0.38958
		$\hat{\gamma}$	0.45253	0.11295	0.71049	0.66100	0.49575	0.53832	0.52994	0.61609
		$\hat{\eta}$	0.08680	0.04165	0.13806	0.13217	0.09634	0.10506	0.10447	0.11760
	MREs	$\hat{\alpha}$	0.09471	0.07236	0.13892	0.13298	0.10248	0.10824	0.10442	0.14167
		$\hat{\gamma}$	0.22627	0.05648	0.35525	0.33050	0.24787	0.26916	0.26497	0.30804
		$\hat{\eta}$	0.12956	0.06216	0.20606	0.19727	0.14379	0.15680	0.15592	0.17552

Table 4. Simulation results of the eight estimation methods for $\varphi = (\alpha = 2.75, \gamma = 0.50, \eta = 0.67)^{\intercal}$

n	Measures	Est. Par.	MLEs	MPSEs	LSEs	CVMEs	WLSEs	PCEs	ADEs	RADEs
20	AVEs	$\hat{\alpha}$	2.91188	2.33929	2.47312	2.71757	2.51818	2.35272	2.63180	3.05570
		$\hat{\gamma}$	0.48842	0.50964	0.49030	0.48550	0.53317	0.50091	0.46894	0.60391
		$\hat{\eta}$	0.68486	0.64113	0.65142	0.67555	0.63721	0.64775	0.68818	0.63010
	MSEs	$\hat{\alpha}$	2.37251	2.02382	3.39494	3.52646	2.64139	2.26891	2.13571	3.82407
		$\hat{\gamma}$	0.21315	0.20787	0.24673	0.24729	0.23629	0.21802	0.21021	0.24687
		$\hat{\eta}$	0.10056	0.10118	0.21452	0.20433	0.14778	0.12048	0.10929	0.16860
	ABs	$\hat{\alpha}$	1.54030	1.42261	1.84254	1.87789	1.62523	1.50629	1.46141	1.95552
		$\hat{\gamma}$	0.46168	0.45593	0.49672	0.49728	0.48610	0.46693	0.45849	0.49686
		$\hat{\eta}$	0.31711	0.31809	0.46316	0.45203	0.38442	0.34710	0.33058	0.41061
	MREs	$\hat{\alpha}$	0.56011	0.51731	0.67001	0.68287	0.59099	0.54774	0.53142	0.71110
		$\hat{\gamma}$	0.92337	0.91185	0.99345	0.99457	0.97219	0.93386	0.91697	0.99371
		$\hat{\eta}$	0.47330	0.47476	0.69128	0.67466	0.57377	0.51807	0.49341	0.61285
50	AVEs	$\hat{\alpha}$	2.76145	2.56168	2.63338	2.70804	2.69128	2.53131	2.72030	2.88928
		$\hat{\gamma}$	0.48356	0.52257	0.49526	0.47591	0.51304	0.49393	0.48685	0.54830
		$\hat{\eta}$	0.67916	0.64465	0.66333	0.68186	0.65888	0.66002	0.68013	0.64884
	MSEs	$\hat{\alpha}$	0.76152	0.76832	1.53361	1.54728	1.14839	0.86837	0.95346	1.70764
		$\hat{\gamma}$	0.09750	0.10075	0.17144	0.17316	0.13807	0.11248	0.12034	0.16009
		$\hat{\eta}$	0.03074	0.03083	0.06862	0.07473	0.04757	0.03707	0.03901	0.05735
	ABs	$\hat{\alpha}$	0.87265	0.87654	1.23839	1.24390	1.07163	0.93186	0.97645	1.30677
		$\hat{\gamma}$	0.31225	0.31741	0.41406	0.41613	0.37157	0.33538	0.34690	0.40011
		$\hat{\eta}$	0.17533	0.17557	0.26195	0.27337	0.21811	0.19253	0.19752	0.23949
	MREs	$\hat{\alpha}$	0.31733	0.31874	0.45032	0.45233	0.38968	0.33886	0.35507	0.47519
		$\hat{\gamma}$	0.62449	0.63483	0.82811	0.83225	0.74314	0.67077	0.69379	0.80022
		$\hat{\eta}$	0.26168	0.26205	0.39097	0.40802	0.32554	0.28737	0.29480	0.35744
100	AVEs	$\hat{\alpha}$	2.77570	2.69832	2.67411	2.77855	2.69000	2.63432	2.72432	2.83505
		$\hat{\gamma}$	0.49933	0.52083	0.50201	0.51091	0.49836	0.50365	0.49589	0.52854
		$\hat{\eta}$	0.67041	0.65280	0.66604	0.66777	0.66851	0.66146	0.67196	0.65873
	MSEs	$\hat{\alpha}$	0.39484	0.39191	0.77864	0.88865	0.53411	0.41497	0.50560	0.84537
		$\hat{\gamma}$	0.05174	0.05438	0.10246	0.11072	0.07282	0.05870	0.06881	0.09371
		$\hat{\eta}$	0.01395	0.01513	0.03251	0.03524	0.02153	0.01641	0.01940	0.02594
	ABs	$\hat{\alpha}$	0.62836	0.62603	0.88241	0.94268	0.73083	0.64418	0.71105	0.91944
		$\hat{\gamma}$	0.22747	0.23319	0.32010	0.33275	0.26986	0.24228	0.26232	0.30613
		$\hat{\eta}$	0.11812	0.12300	0.18030	0.18774	0.14675	0.12811	0.13928	0.16106
	MREs	$\hat{\alpha}$	0.22849	0.22765	0.32088	.34279	0.26575	0.23425	0.25856	0.33434
		$\hat{\gamma}$	0.45494	0.46638	0.64019	0.66549	0.53971	0.48456	0.52464	0.61226
		$\hat{\eta}$	0.17630	0.18358	0.26910	0.28020	0.21903	0.19121	0.20787	0.24039
200	AVEs	$\hat{\alpha}$	2.75642	2.72358	2.73158	2.73308	2.73023	2.70604	2.73049	2.82219
		$\hat{\gamma}$	0.49335	0.50458	0.50950	0.48942	0.50161	0.50812	0.49291	0.52547
		$\hat{\eta}$	0.67401	0.66296	0.66417	0.67759	0.66760	0.66060	0.67270	0.65772
	MSEs	$\hat{\alpha}$	0.18074	0.18477	0.42040	0.42024	0.26755	0.19801	0.24286	0.44068
		$\hat{\gamma}$	0.02579	0.02403	0.05740	0.05471	0.03968	0.02816	0.03420	0.04893
		$\hat{\eta}$	0.00680	0.00656	0.01626	0.01616	0.01043	0.00747	0.00930	0.01217
	ABs	$\hat{\alpha}$	0.42513	0.42985	0.64838	0.64826	0.51725	0.44499	0.49281	0.66384
		$\hat{\gamma}$	0.16058	0.15500	0.23958	0.23390	0.19920	0.16782	0.18493	0.22119
		$\hat{\eta}$	0.08248	0.08101	0.12750	0.12712	0.10215	0.08642	0.09646	0.11030
	MREs	$\hat{\alpha}$	0.15459	0.15631	0.23578	0.23573	0.18809	0.16181	0.17920	0.24140
		$\hat{\gamma}$	0.32116	0.31000	0.47916	0.46781	0.39840	0.33564	0.36987	0.44238
		$\hat{\eta}$	0.12311	0.12092	0.19030	0.18973	0.15246	0.12898	0.14397	0.16463
400	AVEs	$\hat{\alpha}$	2.74072	2.73846	2.72275	2.75357	2.73494	2.71831	2.75870	2.78918
		$\hat{\gamma}$	0.49306	0.50492	0.49426	0.49675	0.49279	0.50363	0.50195	0.51476
		$\hat{\eta}$	0.67526	0.66414	0.67248	0.67189	0.67232	0.66654	0.66959	0.66299
	MSEs	$\hat{\alpha}$	0.09496	0.08605	0.23124	0.22958	0.13180	0.10080	0.12672	0.21980
		$\hat{\gamma}$	0.01368	0.00980	0.03201	0.03110	0.01815	0.01434	0.01825	0.02609
		$\hat{\eta}$	0.00362	0.00267	0.00831	0.00819	0.00468	0.00361	0.00459	0.00619
	ABs	$\hat{\alpha}$	0.30815	0.29334	0.48087	0.47914	0.36304	0.31749	0.35598	0.46883
		$\hat{\gamma}$	0.11694	0.09897	0.17891	0.17635	0.13474	0.11974	0.13508	0.16152
		$\hat{\eta}$	0.06013	0.05171	0.09118	0.09047	0.06843	0.06010	0.06772	0.07867
	MREs	$\hat{\alpha}$	0.11205	0.10667	0.17486	0.17423	0.13201	0.11545	0.12945	0.17048
		$\hat{\gamma}$	0.23389	0.19794	0.35783	0.35270	0.26948	0.23949	0.27015	0.32304
		$\hat{\eta}$	0.08974	0.07718	0.13609	0.13504	0.10213	0.08971	0.10108	0.11742

Table 5. Simulation results of the eight estimation methods for $\varphi = (\alpha = 2.75, \gamma = 0.50, \eta = 1.50)^{\mathsf{T}}$

n	Measures	Est. Par.	MLEs	MPSEs	LSEs	CVMEs	WLSEs	PCEs	ADEs	RADEs
20	AVEs	$\hat{\alpha}$	2.90343	2.41702	2.49703	2.77100	2.45786	2.41480	2.66096	2.97477
		$\hat{\gamma}$	0.51303	0.52863	0.49951	0.48743	0.50168	0.51204	0.47782	0.57411
		$\hat{\eta}$	1.50963	1.40434	1.45476	1.53200	1.46588	1.43369	1.52228	1.42453
	MSEs	$\hat{\alpha}$	2.29599	2.05640	3.30807	3.36388	2.72663	2.20354	2.09744	3.74722
		$\hat{\gamma}$	0.21174	0.20820	0.24700	0.24592	0.23480	0.22037	0.20703	0.24592
		$\hat{\eta}$	0.50532	0.48921	1.02482	1.03259	0.78388	0.57168	0.52528	0.81807
	ABs	$\hat{\alpha}$	1.51525	1.43402	1.81881	1.83409	1.65125	1.48443	1.44826	1.93577
		$\hat{\gamma}$	0.46016	0.45629	0.49699	0.49591	0.48456	0.46944	0.45500	0.49591
		$\hat{\eta}$	0.71086	0.69943	1.01233	1.01616	0.88537	0.75610	0.72476	0.90447
	MREs	$\hat{\alpha}$	0.55100	0.52146	0.66139	0.66694	0.60045	0.53979	0.52664	0.70392
		$\hat{\gamma}$	0.92031	0.91259	0.99397	0.99181	0.96912	0.93887	0.91001	0.99181
		$\hat{\eta}$	0.47390	0.46629	0.67489	0.67744	0.59025	0.50407	0.48318	0.60298
50	AVEs	$\hat{\alpha}$	2.77955	2.56534	2.64386	2.75591	2.65465	2.57022	2.73965	2.83955
		$\hat{\gamma}$	0.49572	0.51715	0.49581	0.49103	0.50758	0.50566	0.49928	0.53963
		$\hat{\eta}$	1.52367	1.45162	1.49119	1.51101	1.47626	1.47207	1.50397	1.45502
	MSEs	$\hat{\alpha}$	0.81967	0.72572	1.56623	1.58154	1.06035	0.86569	0.98967	1.64580
		$\hat{\gamma}$	0.10223	0.09701	0.17341	0.17343	0.13487	0.10754	0.11945	0.16034
		$\hat{\eta}$	0.15631	0.14482	0.35480	0.36260	0.22922	0.17378	0.19277	0.28666
	ABs	$\hat{\alpha}$	0.90536	0.85189	1.25149	1.25759	1.02973	0.93043	0.99482	1.28289
		$\hat{\gamma}$	0.31974	0.31147	0.41643	0.41645	0.36725	0.32793	0.34562	0.40043
		$\hat{\eta}$	0.39536	0.38056	0.59565	0.60216	0.47876	0.41688	0.43906	0.53540
	MREs	$\hat{\alpha}$	0.32922	0.30978	0.45509	0.45731	0.37445	0.33834	0.36175	0.46650
		$\hat{\gamma}$	0.63948	0.62294	0.83285	0.83290	0.73450	0.65586	0.69124	0.80086
		$\hat{\eta}$	0.26357	0.25370	0.39710	0.40144	0.31918	0.27792	0.29271	0.35694
100	AVEs	$\hat{\alpha}$	2.75884	2.65096	2.67985	2.74655	2.70362	2.61822	2.75121	2.83738
		$\hat{\gamma}$	0.48862	0.51175	0.50879	0.50175	0.49765	0.50288	0.49732	0.53902
		$\hat{\eta}$	1.51225	1.46516	1.48695	1.50277	1.49492	1.48220	1.50561	1.46262
	MSEs	$\hat{\alpha}$	0.40105	0.37296	0.80667	0.89662	0.52365	0.41959	0.49604	0.84350
		$\hat{\gamma}$	0.05326	0.04885	0.10198	0.11409	0.07314	0.05913	0.06699	0.09360
		$\hat{\eta}$	0.07629	0.06701	0.15929	0.18465	0.10658	0.08248	0.09744	0.12932
	ABs	$\hat{\alpha}$	0.63329	0.61071	0.89815	0.94690	0.72364	0.64776	0.70430	0.91842
		$\hat{\gamma}$	0.23077	0.22101	0.31935	0.33777	0.27045	0.24317	0.25882	0.30594
		$\hat{\eta}$	0.27621	0.25887	0.39911	0.42971	0.32647	0.28719	0.31216	0.35961
	MREs	$\hat{\alpha}$	0.23029	0.22208	0.32660	0.34433	0.26314	0.23555	0.25611	0.33397
		$\hat{\gamma}$	0.46154	0.44202	0.63869	0.67554	0.54091	0.48634	0.51764	0.61187
		$\hat{\eta}$	0.18414	0.17258	0.26607	0.28647	0.21765	0.19146	0.20811	0.23974
200	AVEs	$\hat{\alpha}$	2.77445	2.71123	2.72260	2.76345	2.69839	2.69544	2.74485	2.76819
		$\hat{\gamma}$	0.50091	0.50907	0.49843	0.50361	0.49287	0.49549	0.50214	0.50091
		$\hat{\eta}$	1.50570	1.48055	1.49749	1.49556	1.50717	1.49709	1.49735	1.49493
	MSEs	$\hat{\alpha}$	0.18004	0.18451	0.43792	0.42650	0.26449	0.19660	0.24555	0.46385
		$\hat{\gamma}$	0.02591	0.02482	0.05983	0.05892	0.03678	0.02979	0.03342	0.05272
		$\hat{\eta}$	0.03415	0.03308	0.08616	0.07948	0.04875	0.03874	0.04337	0.06642
	ABs	$\hat{\alpha}$	0.42431	0.42954	0.66176	0.65307	0.51429	0.44340	0.49553	0.68107
		$\hat{\gamma}$	0.16095	0.15754	0.24460	0.24273	0.19177	0.17259	0.18281	0.22961
		$\hat{\eta}$	0.18479	0.18188	0.29353	0.28192	0.22079	0.19683	0.20826	0.25772
	MREs	$\hat{\alpha}$	0.15430	0.15620	0.24064	0.23748	0.18701	0.16124	0.18019	0.24766
		$\hat{\gamma}$	0.32191	0.31508	0.48920	0.48545	0.38354	0.34518	0.36562	0.45923
		$\hat{\eta}$	0.12319	0.12125	0.19568	0.18795	0.14719	0.13122	0.13884	0.17181
400	AVEs	$\hat{\alpha}$	2.74461	2.73444	2.73143	2.76710	2.73520	2.72389	2.74380	2.76926
		$\hat{\gamma}$	0.49791	0.50565	0.50117	0.50261	0.49341	0.50161	0.50207	0.50469
		$\hat{\eta}$	1.50408	1.48832	1.49534	1.49765	1.50456	1.49305	1.49595	1.49604
	MSEs	$\hat{\alpha}$	0.08983	0.08447	0.21277	0.22661	0.13132	0.10080	0.13266	0.22663
		$\hat{\gamma}$	0.01305	0.00995	0.02933	0.03072	0.01866	0.01369	0.01859	0.02594
		$\hat{\eta}$	0.01691	0.01378	0.03931	0.03941	0.02423	0.01750	0.02412	0.03276
	ABs	$\hat{\alpha}$	0.29971	0.29063	0.46127	0.47604	0.36238	0.31748	0.36423	0.47606
		$\hat{\gamma}$	0.11423	0.09976	0.17125	0.17528	0.13660	0.11700	0.13634	0.16105
		$\hat{\eta}$	0.13005	0.11741	0.19828	0.19852	0.15564	0.13228	0.15530	0.18100
	MREs	$\hat{\alpha}$	0.10899	0.10568	0.16774	0.17310	0.13178	0.11545	0.13245	0.17311
		$\hat{\gamma}$	0.22845	0.19951	0.34249	0.35056	0.27319	0.23399	0.27268	0.32210
		$\hat{\eta}$	0.08670	0.07827	0.13218	0.13235	0.10376	0.08819	0.10353	0.12067

6 Modeling Insurance Data

In this section, we illustrate the applicability and superiority of the T1Ex-W distribution by comparing its goodness of fit to other well-known distributions which are used before for modeling financial and insurance data sets. These distributions include the Weibull, Lomax, Burr, and exponentiated Weibull (EW) distributions.

The analyzed insurance data can be found at this link: https://www.dhs.wisconsi n.gov/stats/index.htm, and it represents hospital cost in the state of Wisconsin provided by the Office of the Health Care Information, Wisconsin's Department of Health and Human Resources.

We calculate certain analytical measures including the Akaike information criterion (AIC), Bayesian information criterion (BIC), Cramer–Von–Messes (CM) statistic, Anderson–Darling (AD) statistic and Kolmogorov–Simonrove (KS) with its related p-value. It is observed that, the TIEx-W distribution gives a close fit to the analyzed data.

The parameters estimates with their standard errors (in parenthesis) are listed in Table **??**. The goodness-of-fit measures are reported in Table **??**. The fitted cdf and Kaplan–Meier survival (KMS) plots are displayed in Figure 2. The PP and box plots are sketched in Figure 3. The plots show that the insurance data has a heavier tail and the TIEx-W distribution fits it very closely.

7 Bayesian Estimation From Insurance Data

In this section, the insurance data is analyzed using the Bayesian analysis. Let the parameters α, γ and η of TIEx-W distribution have independent gamma priors as

$$\alpha \sim Gamma(a,b), \gamma \sim Gamma(c,d), \eta \sim Gamma(e,f),$$

where a, b, c, d, e and f are positive. Then, the joint prior density follows as

$$\pi(\alpha,\gamma,\eta) = \frac{b^a d^c f^e}{\Gamma(a)\Gamma(c)\Gamma(e)} \alpha^{a-1}\gamma^{c-1}\eta^{e-1} e^{-(b\alpha+d\gamma+f\eta)}. \quad (24)$$

We will adopt the well-known five loss functions which are listed Table **??**, with their Bayesian estimators (BEs) and posterior risk. More information can be explored in Calabria and Pulcini (1996).

Now, we derive the posterior probability distribution for a complete data. We define the function φ

$$\varphi(\alpha,\gamma,\eta) = \alpha^{a-1}\gamma^{c-1}\eta^{c-1} e^{-(b\alpha+d\gamma+f\eta)}, \quad \alpha,\gamma,\eta > 0.$$

The joint posterior distribution has the form

$$\pi^*(\alpha,\gamma,\eta|data) \propto \pi(\alpha,\gamma,\eta)L(data). \quad (25)$$

Therefore,the joint posterior density (JPD) of the parameters α, γ and η for complete data is obtained by combining Equation 24 and the likelihood function. Hence, the JPD reduces to

$$\pi^*(\alpha,\gamma,\eta|\underline{x}) = K\varphi(\alpha,\gamma,\eta)\prod_{i=1}^{n} \frac{\alpha\gamma x_i^{\alpha-1} e^{-\gamma x_i^{\alpha}} \left(e^{(1-e^{-\gamma x_i^{\alpha}})} + \eta\right)}{e-\bar{\eta}}, \quad (26)$$

where K is defined by

Table 6. Parameters estimates, standard error (in parenthesis) of the TIEx-W and other competing distributions.

Dist.	α	γ	σ	a
TIEx-W	0.762(0.0450)	1.229(0.4038)	0.1850(0.2100)	
Weibull	0.674(0.0224)	1.918(0.0846)		
Lomax	1.569(0.2076)	0.365(0.0747)		
Burr	2.885(0.1298)	0.813(0.0266)		
EW	1.944(0.4596)	2.564(0.2619)		0.487(0.0633)

Table 7. Analytical measures of the TIEx-W and other competing distributions.

Dist.	AIC	BIC	CM	AD	KS	p-value
TIEx-W	168.800	181.596	0.102	0.983	0.031	0.601
Weibull	171.353	183.884	0.112	0.936	0.035	0.506
Lomax	194.249	202.779	0.464	2.623	0.078	0.002
Burr	171.169	184.699	0.250	1.426	0.045	0.229
EW	170.908	183.7093	0.168	0.997	0.049	0.150

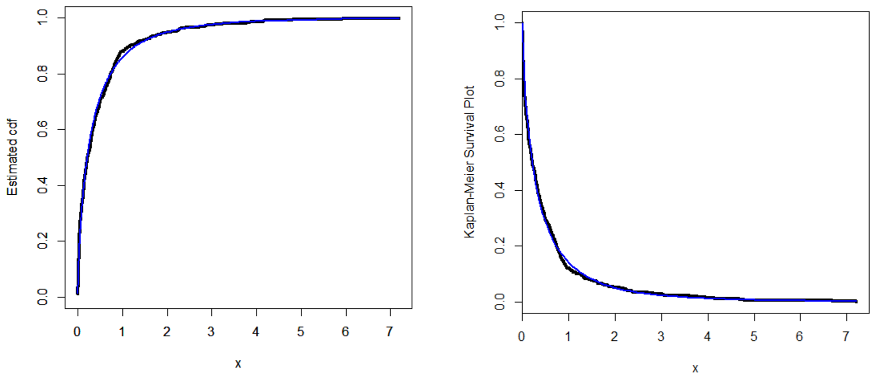

Fig 2. Fitted cdf and KMS plots of the TIEx-W distribution for insurance data.

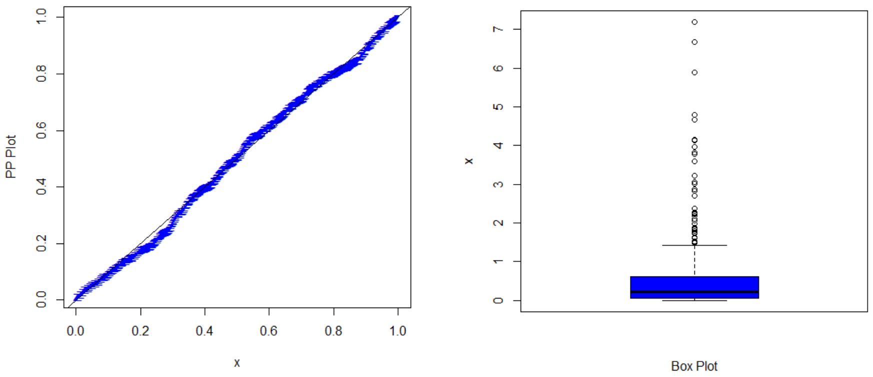

Fig 3. PP plot of the TIEx-W distribution and box plot for insurance data.

$$K^{-1} = \int_0^\infty \int_0^\infty \int_0^\infty \varphi(\alpha,\gamma,\eta) \prod_{i=1}^{n} \frac{\alpha\gamma x_i^{\alpha-1} e^{-\gamma x_i^\alpha} \left(e^{(1-e^{-\gamma x_i^\alpha})} + \eta \right)}{e-\bar{\eta}} d\alpha d\gamma d\eta. \quad (27)$$

$$(28)$$

Equation 26 shows that there is no closed form for the BEs under the loss functions in Table ??, hence we will use the $MCMC$ procedure based on $10,000$ replicates to obtain the BEs. The Bayesian point and interval estimation and posterior risk for the insurance data are listed in Table ??. Table ?? lists 95% credible and HPD intervals for the TIEx-W parameters. The posterior samples are extracted using Gibbs sampling technique. Moreover, the posterior summary plots are provided in Figures 46.

Table 8. Five loss functions and their BEs and posterior risk.

loss function	BEs	Posterior risk
$L_1 = SELF = (\theta - d)^2$	$E(\theta\|x)$	$Var(\theta\|x)$
$L_2 = WSELF = \frac{(\theta-d)^2}{\theta}$	$(E(\theta^{-1}\|x))^{-1}$	$E(\theta\|x) - (E(\theta^{-1}\|x))^{-1}$
$L_3 = MSELF = \left(1 - \frac{d}{\theta}\right)^2$	$\frac{E(\theta^{-1}\|x)}{E(\theta^{-2}\|x)}$	$1 - \frac{E(\theta^{-1}\|x)^2}{E(\theta^{-2}\|x)}$
$L_4 = KLF = \left(\sqrt{\frac{d}{\theta}} - \sqrt{\frac{\theta}{d}}\right)$	$\sqrt{\frac{E(\theta\|x)}{E(\theta^{-1}\|x)}}$	$2\left(\sqrt{E(\theta\|x)E(\theta^{-1}\|x)} - 1\right)$
$L_5 = PLF = \frac{(\theta-d)^2}{d}$	$\sqrt{E(\theta^2\|x)}$	$2\left(\sqrt{E(\theta^2\|x)} - E(\theta\|x)\right)$

Table 9. The BEs and posterior risks of the TIEx-W the parameters for Insurance data

Data	Insurance data					
Bayes	$\widehat{\alpha}$		$\widehat{\gamma}$		$\widehat{\eta}$	
Loss functions	Estimate	Risk	Estimate	Risk	Estimate	Risk
SELF	0.6889	0.0020	0.0174	0.00003	0.0657	0.0010
WSELF	0.6860	0.0030	0.0156	0.0017	0.0420	0.0237
MSELF	0.6829	0.0045	0.0142	0.0928	0.0186	0.5559
PLF	0.6905	0.0029	0.0183	0.0019	0.0733	0.0152
KLF	0.6875	0.0044	0.0165	0.1069	0.0525	0.5026

Table 10. Credible and HPD intervals of the TIEx-W parameters α, γ and η for insurance data.

	Credible interval	HPD interval
α	(0.6569, 0.7224)	(0.5967, 0.7674)
γ	(0.01357, 0.02073)	(0.0085, 0.0299)
η	(0.04710 0.07481)	(0.0077, 0.1370)

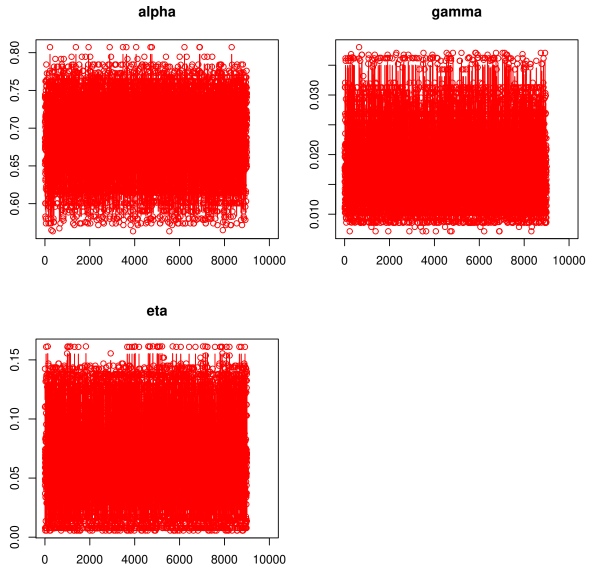

Fig 4. Trace plots of the TIEx-W parameters for insurance data based on Gibbs sampling.

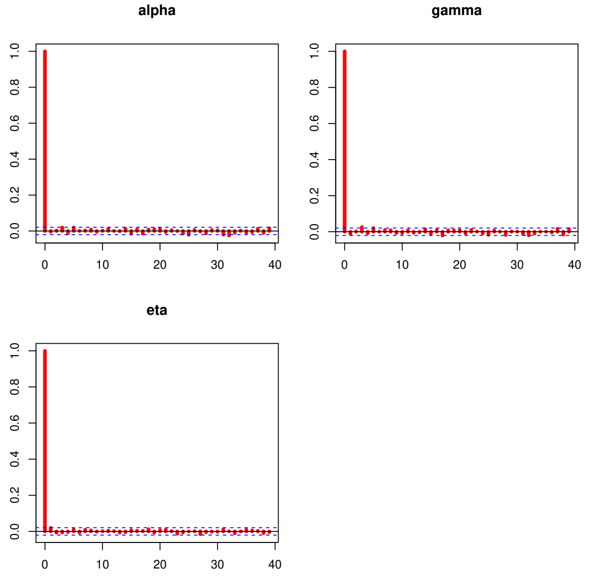

Fig 5. Autocorrelation plots of the TIEx-W parameters for insurance data based on Gibbs sampling.

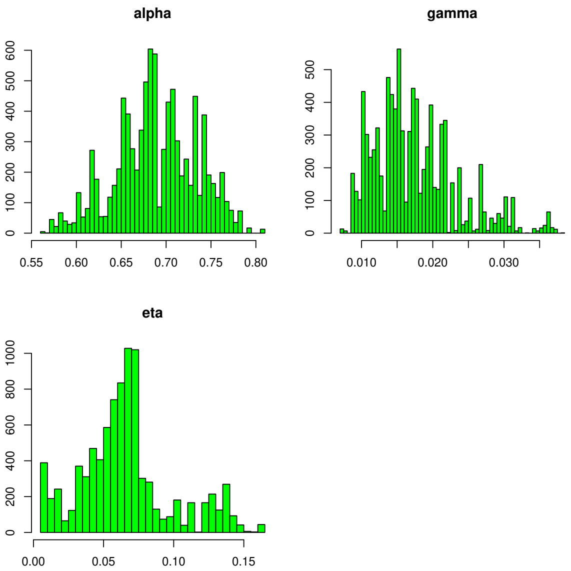

Fig 6. Histogram plots of the TIEx-W parameters for insurance data based on Gibbs sampling.

8 Concluding Remarks

In the present paper, we have introduced a new class of heavy-tailed distributions allowing closed form expressions for distribution function and some of its basic properties. The proposed class is called type-I extended-F (TIEx-F) family and one of its special sub-models, TIEx-Weibull (TIEx-W), is addressed. The model parameters are obtained based on eight methods of estimation, and a detailed simulation study is provided. Bayesian analysis based on insurance data is also discussed. The applicability of the TIEx-W distribution has been illustrated using an insurance data set.

Funding: This study was funded by the Taif University Researchers Supporting Project (TURSP-2020/316), Taif University, Taif, Saudi Arabia.

Conflicts of Interest: The authors declare no conflict of interest.

References

1. Afify, A.Z., Al-Mofleh, H., Aljohani, H.M., & Cordeiro, G.M. (2022). The Marshall–Olkin–Weibull-H family: estimation, simulations, and applications to COVID-19 data. Journal of King Saud University-Science, 34, p.102115.

2. Ahmad, Z., Ilyas, M., & Hamedani, G.G. (2019). The Extended Alpha Power Transformed Family of Distributions: Properties and Applications. Journal of Data Science, 17, 726-741.

3. Al-Babtain, A.A., Kumar, D., Gemeay, A.M., Dey, S., & Afify, A.Z. (2021). Modeling engineering data using extended power-Lindley distribution: Properties and estimation methods. Journal of King Saud University-Science, 33, p.101582.

4. Ampadu, C.B. (2019). The Ampadu APT qTX - Family of Distributions Induced by V with an Illustration to Data in the Health Sciences. Annals of Biostatistics & Biometric Applications, 2, 1-5.

5. Bakar, S.A., Hamzah, N.A., Maghsoudi, M., & Nadarajah, S. (2015). Modeling loss data using composite models. Insurance: Mathematics and Economics, 61, 146-154.

6. Bhati, D., & Ravi, S. (2018). On generalized log-Moyal distribution: A new heavy tailed size distribution. Insurance: Mathematics and Economics, 79, 247-259.

7. Calabria, R., & Pulcini, G. (1996). Point estimation under asymmetric loss functions for left-truncated exponential samples. Communications in Statistics-Theory and Methods, 25, 585-600.

8. Calderín-Ojeda, E., & Kwok, C.F. (2016). Modeling claims data with composite Stoppa models. Scandinavian Actuarial Journal, 2016, 817-836.

9. Cooray, K., & Ananda, M.M. (2005). Modeling actuarial data with a composite lognormal-Pareto model. Scandinavian Actuarial Journal, 2005, 321-334.

10. Coronel-Brizio, H.F., & Hernandez-Montoya, A.R. (2005). On fitting the Pareto–Levy distribution to stock market index data: selecting a suitable cutoff value. Physica A: Statistical Mechanics and its Applications, 354, 437-449.

11. Ghitany, M., Gómez-Déniz, E., & Nadarajah, S. (2018). A New Generalization of the Pareto Distribution and Its Application to Insurance Data. Journal of Risk and Financial Management, 11, 10.

12. Klugman, S.A., Panjer, H.H., & Willmot, G.E. (2012). Loss models: from data to decisions (Vol. 715). John Wiley & Sons.

13. Mahdavi, A., & Kundu, D. (2017). A new method for generating distributions with an application to exponential distribution. Communications in Statistics-Theory and Methods, 46, 6543-6557.

14. McNeil, A. J. (1997). Estimating the tails of loss severity distributions using extreme value theory. ASTIN Bulletin: The Journal of the IAA, 27(1), 117-137.

15. Mead, M.E., Cordeiro, G. M., Afify, A.Z., & Al-Mofleh, H. (2019). The alpha power transformation family: properties and applications. Pak. j. stat. oper. res., 15, 525-545.

16. Nadarajah, S., & Bakar, S.A. (2014). New composite models for the Danish fire insurance data. Scandinavian Actuarial Journal, 2014, 180-187.

17. Resnick, S.I. (1997). Discussion of the Danish data on large fire insurance losses. ASTIN Bulletin: The Journal of the IAA, 27, 139-151.

18. Scollnik, D.P., & Sun, C. (2012). Modeling with Weibull–Pareto models. North American Actuarial Journal, 16, 260-272.
