# Supplementary material for: Classical and Bayesian estimation for type-I extended-F family with an actuarial application
Source: PLoS One. 2023 Feb 2;18(2):e0275430. doi: 10.1371/journal.pone.0275430 (PMC9894468; doi:10.1371/journal.pone.0275430)
Supplement: Attachment — Submitted filename: Review Report for PONE-D-22-21984.docx [file pone.0275430.s001.docx]

Review Report for PONE-D-22-21984

This study proposes a new family of distributions named Type-I Extended-F Family (TIEx-F) with a specific focus on modelling insurance data. Some properties and numerous classical and Bayesian estimation approaches are introduced for estimating the parameters of a special case, the TIEx-Weibull (TIEx-W) model. Finally, the author demonstrate the potentials of the model by applying it to a single insurance datasets. The authors have performed a good job, however, I have some issues and suggestions that may enhance the current version of the manuscript.

1. There should be more clear motivation of the study.
2. The authors refers the TIExF family "the most tractable for simple analytical expressions" with TIEx-W as a special case, I expect the authors to assess the TIEx-W with other competing non-standard distributions, such as the special cases derived from families defined in Equations (1)-(3). Going by this will explore more the potentials of the proposed family.
3. Check the correctness of the sentence and citations in page 3, lines 55-56.
4. The authors should provide the other basic functions of the TIEx-W distribution, including the survival and hazard rate functions, along with plot(s) showing some possible shapes of the hazard rate function for different settings of the model's parameters. Also present some discussions on the plots and whether the hazard rate function is relevant in modeling insurance data.
5. In section 3, more properties of the distribution should be present not only the quantile function and moments.
6. Do your quantile function has a closed form expression? If yes, please, write it clearly, and if no, provide a procedure on how to draw random sample from the quantile function.
7. Table numbers are missing in the text. Check and correct it.
8. Check and correct the third term of Eq. (15). Not correctly differentiated.
9. In section 5, I suggest the authors to include simulation results for the Bayesian approach under the selected loss functions to enable the comparison of all the proposed estimators for the TIEx-W model.
10. In section 7, discuss the reasons/justifications for choosing gamma priors for the three parameters f TIEx-W model.
11. The authors need to provide some discussions on the Bayesian results.
12. I also expect to see some comparisons between the results from the classical estimation methods and the Bayesian results either by plotting the density curves, CDF curves for the trained TIEx-W model from all the estimation methods employed or the p-p plots. This is essential to identify the best estimation method(s) in solving real-world problems.
13. How the estimated values of the parameters can be translated in the real life situations?

Lastly, the manuscript may be considered for publishing in PONE if the concerns raised are addressed thoroughly.
